# Product Inhibition and pH Affect Stoichiometry and Kinetics of Chain Elongating Microbial Communities in Sequencing Batch Bioreactors

**DOI:** 10.3389/fbioe.2021.693030

**Published:** 2021-06-21

**Authors:** Maximilienne Toetie Allaart, Gerben Roelandt Stouten, Diana Z. Sousa, Robbert Kleerebezem

**Affiliations:** ^1^Department of Biotechnology, Delft University of Technology, Delft, Netherlands; ^2^Laboratory of Microbiology, Wageningen University & Research, Wageningen, Netherlands

**Keywords:** fermentation, reversed beta-oxidation, anaerobic, caproate, butyrate, product inhibition, enrichment, *Clostridium kluyveri*

## Abstract

Anaerobic microbial communities can produce carboxylic acids of medium chain length (e.g., caproate, caprylate) by elongating short chain fatty acids through reversed β-oxidation. Ethanol is a common electron donor for this process. The influence of environmental conditions on the stoichiometry and kinetics of ethanol-based chain elongation remains elusive. Here, a sequencing batch bioreactor setup with high-resolution off-gas measurements was used to identify the physiological characteristics of chain elongating microbial communities enriched on acetate and ethanol at pH 7.0 ± 0.2 and 5.5 ± 0.2. Operation at both pH-values led to the development of communities that were highly enriched (>50%, based on 16S rRNA gene amplicon sequencing) in *Clostridium kluyveri* related species. At both pH-values, stably performing cultures were characterized by incomplete substrate conversion and decreasing biomass-specific hydrogen production rates during an operational cycle. The process stoichiometries obtained at both pH-values were different: at pH 7.0, 71 ± 6% of the consumed electrons were converted to caproate, compared to only 30 ± 5% at pH 5.5. Operating at pH 5.5 led to a decrease in the biomass yield, but a significant increase in the biomass-specific substrate uptake rate, suggesting that the organisms employ catabolic overcapacity to deal with energy losses associated to product inhibition. These results highlight that chain elongating conversions rely on a delicate balance between substrate uptake- and product inhibition kinetics.

## Introduction

In the transition from a petroleum-based to a bio-based society, the microbial production of chemicals from renewable resources has gained significant interest. Medium-chain carboxylic acids (MCCAs) are an example of native microbial products that can be generated from (complex) substrates, ranging from organic to gaseous waste streams (Steinbusch et al., 2011; Agler et al., 2012; Ge et al., 2015; Diender et al., 2016, 2019; Ganigué et al., 2016). Caproic acid, a six-carbon MCCA can be used as antimicrobial agent replacing antibiotics in animal feed (Desbois, 2012), as corrosion inhibitor, and as precursor for flavors and fragrances, solvents and fuels (Agler et al., 2011).

Caproic acid is naturally produced in different types of microbial ecosystems (Barker and Taha, 1942; Weimer and Stevenson, 2012; Candry et al., 2020) by elongating short-chain carboxylic acids using ethanol or lactate as electron donor through reversed β-oxidation (Spirito et al., 2014). Chain elongating organisms compete for substrate with acetoclastic methanogens, syntrophic ethanol oxidizers and sulfate reducing organisms and provide substrate to, for example, homoacetogens, syntrophic butyrate oxidizing organisms and hydrogenotrophic methanogens (Muyzer and Stams, 2008; Cavalcante et al., 2017). By tuning the operational parameters in a bioreactor, the competition between these organisms can be controlled. In this way, mixed microbial communities of chain elongators can be enriched in non-sterile environments (Kleerebezem and van Loosdrecht, 2007).

The biochemistry of the model organism for chain elongation, *Clostridium kluyveri*, was unraveled using whole-genome sequencing (Seedorf et al., 2008) and efforts were made to quantify the kinetic properties of this organism (Candry et al., 2018). Thermodynamic analyses (Angenent et al., 2016; Cavalcante et al., 2017) and experiments with different electron donors and acceptors in different ratios (Kenealy and Waselefsky, 1985; Spirito et al., 2018) were performed to shed light on the stoichiometry of the process and the flexibility of chain elongating metabolism. Despite these efforts, several aspects of the quantitative description of the chain elongation process remain to be elucidated. For example, the effect of pH on inhibition by weak acids is not reflected in the available kinetic model, nor is the effect of different electron donor versus electron acceptor ratios on the observed stoichiometries and rates. All in all, various of the fundamental aspects of chain elongation remain to be uncovered.

Fundamental insights are key in overcoming the challenges, such as steering the product spectrum and efficiently balancing integrated production- and extraction processes, in the exploitation of chain elongating organisms for the production of biochemicals. Nevertheless, a large part of the scientific literature published in recent years has been focused on process intensification (Grootscholten et al., 2013; Kucek et al., 2016; Spirito et al., 2018) and the application of chain elongation in the conversion of real waste streams (Vasudevan et al., 2014; Gildemyn et al., 2017; Wu et al., 2020). This focus on the overall process results in the analysis of conversions at the reactor level, and the effects on the kinetic properties of the microorganisms involved are therefore often overlooked in such systems. Therefore, an experimental setup in which process parameters and microbial conversions can be closely monitored and controlled is required to generate a comprehensive image of microbial behavior.

We have successfully operated sequencing batch bioreactors (SBRs) with a suspended mixed microbial community converting acetate and ethanol to butyrate and caproate at pH 7.0 and 5.5. With these experiments, we gained insight in the effects of product inhibition on chain elongating microorganisms. These findings pave the way toward in-depth characterization and understanding of the microbial physiology of chain elongators, which in turn increases the extent to which we can control mixed culture fermentations toward MCCAs.

## Materials and Methods

### Reactor Operation

The enrichments were carried out in glass jacketed 2 L bioreactors (Applikon, Netherlands) with a working volume of 1 L. The bioreactors were operated in sequencing batch mode, with a cycle time of 12 h. Each cycle 50% of the volume of the bioreactor was exchanged for 50 g of nutrient stock solution and 400 g of demineralized water. One hour after the start of the effluent phase a 50 g pulse of carbon solution was fed, dosing 125.5 mmol ethanol and 21.1 mmol acetate to the reactor in each cycle. This equaled an ethanol-to-acetate ratio of 6:1 in the bioreactor feed. A relatively high concentration of ethanol versus acetate was reported to positively influence caproate production (Liu et al., 2016) and a 6:1 ratio enabled both butyrate and caproate formation at pH 5.5 and pH 7.0 (Candry et al., 2020). Therefore, this feeding regime was chosen to allow for the characterization of the effect of both butyrate and caproate on chain elongating communities at different pH values. Anaerobic conditions were ensured by continuously sparging the reactors with a mixture N_2_ and CO_2_ (95:5 vol%) at a rate of 46.6 mL min^–1^ (MX44, DASGIP, Germany). As CO_2_ is required for growth of *C. kluyveri* (Tomlinson and Barker, 1954), CO_2_ was continuously sparged through the culture broth in non-limiting amounts. The headspace gas was continuously recirculated through the broth using a gas recirculation pump. To improve the quality of the gas data, the reactors were operated at 0.1 bar overpressure. The suspended culture was taken out of the reactors weekly for biofilm removal from the reactor walls. The reactors were continuously agitated at a speed of 400 rpm (SC4, DASGIP, Germany) using mechanical stirrers. Reactor temperature was maintained at 34°C using a water jacket and a thermostat bath (E300, Lauda, Germany). To prevent culture broth evaporation, the off-gas was cooled using a cryostat set to 5°C. Reactor pH was maintained at 5.5 ± 0.2 or 7.0 ± 0.2 using 1 mol L^–1^ NaOH and 1 mol L^–1^ HCl solutions. The pumps for feeding, effluent removal and pH control were controlled as described elsewhere, as well as the methods for data acquisition (Stouten et al., 2019). The reactors were inoculated with a chain elongating community enriched from anaerobic digester sludge (Harnaschpolder, Netherlands) for approximately three months with the same feeding regime and the same enrichment medium as described here. During the enrichment of the inoculum for the bioreactors operated here, the pH could not be controlled at a single value due to electrical interference with the pH measurement. This led to a pH fluctuating between values of approximately 5–7.5 in the initial enrichment stage.

### Enrichment Medium

An adapted *Clostridium kluyveri* medium was used as enrichment medium. The carbon- and nutrient source solutions were prepared separately as 10× concentrated stocks. The carbon stock solution contained 2.6 M ethanol and 0.44 M potassium acetate and the nutrients stock contained 67.6 mM KH_2_PO_4_, 93.5 mM NH_4_Cl, 4.1 mM MgSO_4_⋅7 H_2_O, 5.9 mM MgCl_2_⋅6 H_2_O, 20 mL of alkaline trace elements solution, 20 mL of SL-10 trace elements solution and 20 mL of B vitamin solution. The alkaline trace elements solution contained (in g L^–1^): NaOH 0.4, Na_2_SeO3 0.017, Na_2_WO_4_⋅2 H_2_O 1.03, Na_2_MoO_4_.2H2O 0.024. The SL-10 trace elements solution contained (in g L^–1^): FeCl_2_⋅4 H_2_O 1.5, FeCl_3_⋅6 H_2_O 2.5, ZnCl_2_ 0.07, MnCl_2_ ⋅4 H_2_O 0.1, H_3_BO_3_ 0.006, CoCl_2_ ⋅6 H_2_O 0.19, CuCl_2_ ⋅2 H_2_O 0.002, NiCl_2_ ⋅6 H_2_O 0.024, Na_2_MoO_4_ ⋅2 H_2_O 0.036 and 10 mL 25% HCl. The B vitamin solution contained (in g L^–1^): biotin 0.02, nicotinamide 0.2, *p*-aminobenzoic acid 0.1, thiamine hydrochloride 0.2, Ca-pantothenate 0.1, pyridoxamine 0.5, cyanocobalamine 0.1, riboflavin 0.1. From cycle 45 onward, 50 mM 2-bromoethanesulfonate (BES) (TCI, Tokyo, Japan) was added to the 10x concentrated medium.

### Analytical Methods

The in- and off-gas composition of the reactors were measured using mass spectrometry (PRIMA BT Benchtop, Thermo Fisher Scientific, United Kingdom). pH values were stored and acid- and base dosage were monitored using an integrated revolution counter (MP8, DASGIP, Germany). Biomass concentrations were monitored both by measuring optical density (OD_660_) and the amount of VSS in the broth using 150 mL effluent (APHA/AWWA/WEF, 1999), calculated assuming a biomass molecular weight of 24.6 g Cmol^–1^. Technical replicates of both OD and VSS measurements were always performed. End of cycle acetate concentrations were determined using high performance liquid chromatography (HPLC) using an Aminex HPX-87H column (BioRad, United States) at 59°C coupled to an ultraviolet detector at 210 nm (Waters, United States). 1.5 mmol L^–1^ phosphoric acid was used as eluent. Biomass was removed from the reactor samples by centrifugation and filtration using a 0.22 μm membrane filter (Millipore, Millex-GV, Ireland). Ethanol, butyrate and caproate concentrations were analyzed using gas chromatography (GC). GC was performed using a Trace 1300 machine (Thermo Fisher Scientific, United States) equipped with an injector maintained at 180°C, a carbowax polyethylene glycol column of 20 m × 0.18 mm (Agilent, United States), using a temperature gradient from 50 to 180°C over 24 min. Helium was used as carrier gas and fermentation substrates and products were detected using a flame ionization detector set at 200°C. Iso-hexanoic acid was used as internal standard and samples were acidified using pure formic acid (Sigma Aldrich, United States). The analytical methods allowed for the measurement of longer- and odd-chain carboxylic acids, but these were not detected.

### Microbial Community Analysis

The microbial community composition was analyzed using cell pellets of 4 mL samples taken at the end of a cycle. DNA was extracted from the cell pellets from different time points in the enrichment using the PowerSoil microbial extraction kit (Qiagen Inc., Germany) following manufacturer’s instructions. The DNA-content of the extracts was quantified using a Qubit 4 (Thermo Fisher Scientific, United States) to confirm sufficient DNA was extracted for analysis. The samples were sent to Novogene Ltd. (Hongkong, China) for amplicon sequencing of the V3-V4 region of the 16S-rRNA gene (position 341–806) on an Illumina paired-end platform. After sequencing, the raw reads were quality filtered, chimeric sequences were removed and OTUs were generated on the base of ≥97% identity. Subsequently, the representative sequence for each OTU was annotated using the GreenGene Database. The sequences have been stored in the 4TU research database and can be found under the DOI 10.4121/14394578.

### Parameter Estimation

The carbon- and electron balances were calculated using the gas productivities and end of cycle product and residual substrate concentrations. As the SBRs were operated at an exchange ratio of 0.5, and in case of steady state operational performance, 50% of the substrates and products amounts present at the end of a cycle correspond to the initial amounts of the next cycle (steady state assumption).

The average biomass, caproate and hydrogen yields on ethanol were calculated using the amount of ethanol consumed and the amount of biomass (according to VSS measurements), caproate or hydrogen produced in a cycle. As 50% of the culture broth is removed every 12 h, the biomass has to duplicate within this time during stable bioreactor operation. Therefore, the average growth rate (μ) during one cycle can be estimated directly using the steady assumption, the exchange ratio and the cycle time. From the average growth rate and the biomass yield on ethanol ${\tilde{(Y}}_{X/EtOH})$ the average biomass specific ethanol uptake rate (${\tilde{q}}_{EtOH}$) can directly be estimated according to ${\tilde{q}}_{EtOH}=\tilde{\mu }/{\tilde{Y}}_{X/EtOH}$. The standard deviations in the calculated ${\tilde{q}}_{EOH}$ values were determined with error propagation of the standard deviation in the measured biomass- and ethanol concentrations.

### Modeling Bioreactor-Specific Gas Productivity

The actual bioreactor-specific respiration rates of H_2_, N_2_, CO_2_, and CH_4_ were reconstructed from the off-gas composition measurements by modeling them in a physicochemical model that mimics the operated bioreactors. In order to do this, a Particle Filter state estimation method (Stouten et al., 2021) was used. The main physicochemical processes, which include inorganic carbon speciation, gas mixing in the bulk liquid and head space, delays and non-equilibrium phase transfer rates, are incorporated in a model that allows for rate estimation using measured off-gas and pH data for calibration of the modeled respiration rates. For details, see Stouten et al. (2021) and Supplementary Materials.

## Results

### Development of Microbial Community Structure and Functionality

Two bioreactors were operated for 48 days in sequencing batch mode at pH 7.0 ± 0.2 and pH 5.5 ± 0.2. In each operational cycle of 12 h, a substrate pulse of 125 mmol ethanol and 21 mmol acetate was dosed. Both reactors were inoculated with a culture that already exhibited chain elongating activity. Chain elongating activity is associated with hydrogen formation and the consumption of small amounts of CO_2_. The composition of the off-gas and dosage of base were analyzed on-line, which allowed for continuous monitoring of hydrogen and methane formation and carbon dioxide consumption. Furthermore, dissolved substrate and product concentrations at the end of an operational cycle were frequently measured throughout the enrichment to determine the change in product spectrum and extent of substrate consumption.

**FIGURE 1:**
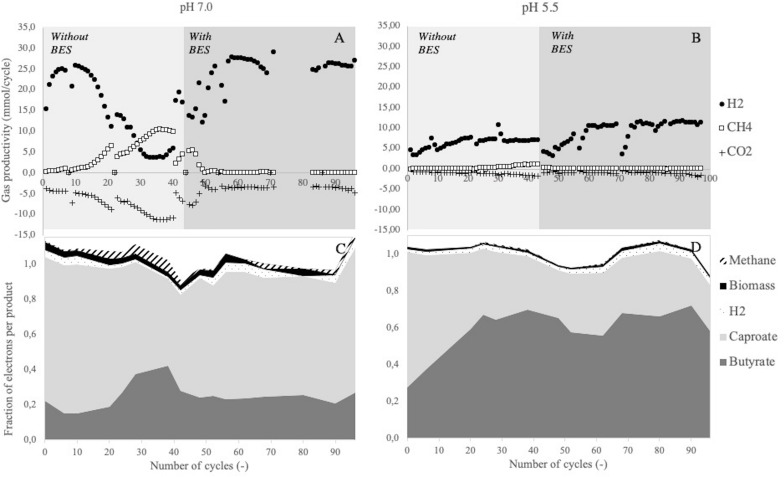
Gas productivities (mmol) per cycle **(A,B)** and distribution of consumed electrons over the fermentation products **(C,D)** over time in SBRs at different pH. A single spike of BES was given to the enrichment at pH 7.0 in cycle 40, from cycle 45 onward both enrichments were continuously supplemented with BES. Due to a technical error, gas data of the enrichment at pH 7.0 is missing between cycle 72 and 82. Sudden drops in hydrogen production at pH 5.5 are due to reactor cleaning, after which the biological activity recovers within 4 cycles.

In both reactors, hydrogen was detected in the off-gas and butyrate and caproate were measured in the liquid product spectrum (Figure 1). This confirmed that chain elongating conversions took place in both conditions. Beside hydrogen, methane was also produced in the initial phase of both enrichments without BES addition (Figures 1A,B), indicating that methane-producing biological conversions took place alongside chain elongation. A simultaneous decrease in net hydrogen productivity and increase in methane productivity and CO_2_ consumption was observed at pH 7.0. This showed that at least a part of the methane production originates from hydrogenotrophic methanogenesis. At pH 5.5, the simultaneous slight increase in CO_2_ consumption and methane production also suggested the presence of hydrogenotrophic methanogens. In order to solely study the characteristics of chain elongation and ensure comparability between the two systems, methanogens were inhibited in both reactors from cycle 45 onward by adding 5 mM of BES to the medium. Gas productivities stabilized within 10 cycles after BES addition and methane production was effectively suppressed (Figures 1A,B). From cycle 60 onward the fractions of butyrate and caproate also stabilized (Figures 1C,D). This confirmed a steady functional performance of the microbial community, consisting of only chain elongating conversions.

**FIGURE 2:**
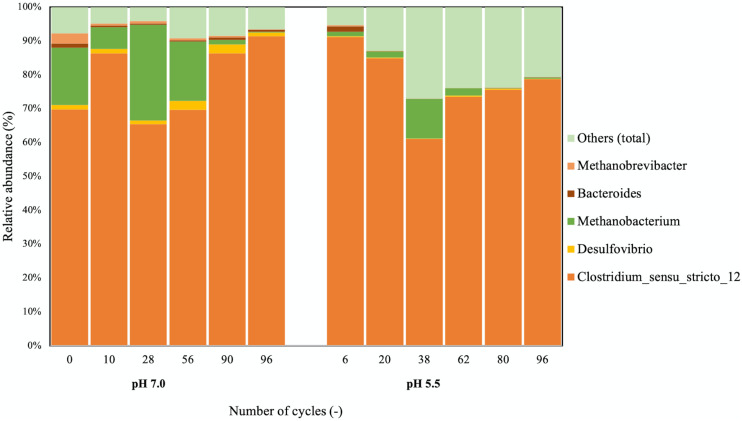
Taxonomic distribution of the microbial communities at pH 7.0 and 5.5. 16S rRNA amplicon sequencing was used to assess the community composition. All samples had a similar number of total reads. OTUs constituting less than 3% of the community were grouped under “Others.” On the x-axis, the number of cycles after which the sample was taken from the bioreactor is displayed for each sample.

16S rRNA gene amplicon sequencing data showed that *Clostridium sensu strictu 12* was the predominant genus at both pH (Figure 2). OTUs of >97% identity with the *C. kluyveri* 16S rRNA gene sequence consequently constituted more than 50% of the total number of reads and *C. kluyveri* was the only species of the *Clostridium* genus that was identified in the microbial community. This indicated that strong enrichment of *C. kluyveri* was maintained in both bioreactors. The presence of *Methanobacterium* coincided with periods of methane formation at both pH-values.

### Stoichiometric and Kinetic Properties of Chain Elongating Communities at Different pH

Stably performing chain elongating communities were established at both pH-values from cycle 60 onward. In both enrichments, high residual ethanol concentrations at the end of the cycle were observed. Residual acetate was also measured in both conditions (Table 1 and Supplementary Table 1). This showed that high residual ethanol concentrations were not related to acetate limitation. At pH 7.0, the consumed acetate and ethanol were converted to mainly caproate, comprising 71 ± 6% of the electrons found in fermentation products (Table 1). Enriching at a pH of 5.5 led to a butyrate-dominated product spectrum (Figure 1D and Table 1).

**TABLE 1:** Average stoichiometric and kinetic properties of chain elongating communities in SBRs at different pH during stable operation^*a*^.

	pH 7	pH 5.5
Ethanol consumption (%)	47 ± 4	25 ± 3
Acetate consumption (%)	79 ± 3	66 ± 4
Electrons converted to caproate (%)	71 ± 6	30 ± 5
EtOH:H_2_ ratio (mol mol^–1^)	3.0 ± 0.3	3.5 ± 0.2
Donor:Acceptor consumption ratio^*b*^ (mol mol^–1^)	1.9 ± 0.1	2.1 ± 0.2
Y_*X/EtOH*_ (Cmol_*X*_ mol^–1^)	0.11 ± 0.02	0.05 ± 0.01
q_*EtOH*_ (mol Cmol_*X*_^–1^ h^–1^)	0.54 ± 0.08	1.12 ± 0.13

*^a^Only data between cycle 60 and cycle 96 is included in these calculations. ^b^Electron acceptor consumption was calculated as the summed consumption of acetate and butyrate. Following the canonical stoichiometry for chain elongation, it was assumed that for every mol of caproate produced, 1 mol of butyrate was consumed.*

Chain elongating organisms couple the oxidation of ethanol to acetate to the reductive elongation of acids in the reversed β-oxidation. The electron bifurcating reduction of crotonyl-CoA to butyryl-CoA discursively allows the release of the electrons originating from ethanol as hydrogen. Therefore, the ethanol-to-hydrogen ratio is a proxy for the metabolic proportion between ethanol oxidation and reversed β-oxidation through which metabolic flexibility can be identified. The canonical stoichiometry, in which 6 ethanol and 4 acetate are converted to 5 butyrate and 2 hydrogen, assumes a ratio of 1:5 between ethanol oxidation and reversed β-oxidation, resulting in the consumption of 3 ethanol per hydrogen formed. At pH 7.0, this canonical stoichiometry was observed (Table 1). At pH 5.5, 3.5 ± 0.2 mol of ethanol were consumed per hydrogen formed. Concomitantly, the relative consumption of electron donor over electron acceptor increased to 2.1 ± 0.2 mol electron donor per mol electron acceptor at pH 5.5 (Table 1). The consumption ratios of electron donor over electron acceptor are in the range of the ratios observed in acetate-limited chemostats of *C. kluyveri* (1.7–2.3 mol mol^–1^) by Kenealy and Waselefsky (1985). Changes in the ethanol-to-hydrogen ratio confirm that the stoichiometry of chain elongation is not fixed (Angenent et al., 2016).

The average growth rate in both bioreactors was 0.058 h^–1^, as imposed by the exchange ratio of 0.5 and the cycle time of 12 h. With this average growth rate and the initial and final substrate- and biomass concentrations (Supplementary Table 1), the biomass yield on ethanol and the average biomass-specific ethanol uptake rate could be calculated. The culture at pH 5.5 exhibited a lower biomass yield on ethanol, but a significantly higher biomass-specific substrate uptake rate (Table 1). This indicates that the substrate requirements for maintenance are higher at low pH.

### On-Line Gas Analysis for Kinetic Bottleneck Identification

**FIGURE 3:**
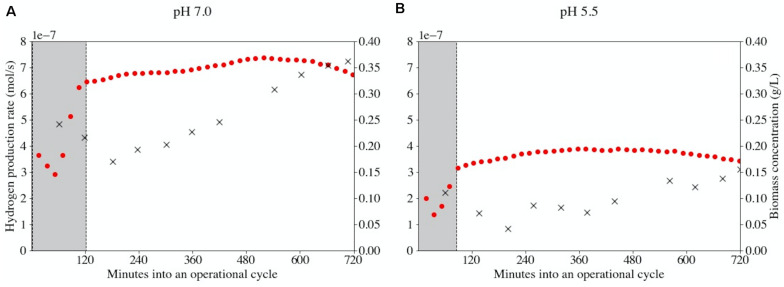
Bioreactor-specific hydrogen production rates (•) and biomass concentrations (×) at pH 7.0 **(A)** and pH 5.5 **(B)** during operational cycle 92. The physicochemical (abiotic) behavior of the reactor was modeled in order to accurately predict the biological activity responsible for the observed gas profiles. Areas shaded in gray indicate the initiation of a new cycle and its associated uncertainty in measurements due to a temporarily changing physiochemistry. This uncertainty is mainly related to incorrect prediction of CO_2_ speciation. The period of uncertainty is shorter at pH 5.5 (90 min versus 120 min) as less CO_2_ dissolves at this pH.

The hydrogen concentration in the bioreactor off-gas is a measure of the biomass-specific catabolic rate if the catabolic stoichiometry is fixed. Therefore, the off-gas measurements were used in combination with a Particle Filter state estimation to calculate the overall hydrogen production rate in the two bioreactors over time. The Particle Filter could reconstruct the hydrogen production rates except for the period of volume exchange (Supplementary Figure 1). In Figure 3 the biomass concentrations and hydrogen production rates during a representative operational cycle (cycle 92) are presented. At this time the reactor performance was stable and biomass duplicated during each operational cycle at both pH-values. The overall hydrogen production rate did not double accordingly. In fact, a near-horizontal profile was obtained in the hydrogen production rate in both conditions. It was ruled out that nutrient limitation caused this behavior (data not shown). Thus, the biomass-specific catabolic flux decreases approximately 50% throughout an operational cycle both at pH 7.0 and at pH 5.5. This indicates that the ratio between substrate and product has a pronounced effect on the biomass-specific rate.

## Discussion

### Enrichment of Chain Elongating Microbial Communities in Sequencing Batch Bioreactors

In this work we have shown that SBR cultivation is suitable for enriching chain elongating communities at pH 5.5 and pH 7.0. Although taxonomically similar microbial communities developed in both enrichments, significant differences were observed in microbial functionality. In both bioreactors, an average growth rate of 0.058 h^–1^ was imposed by the cycle length and exchange ratio. This growth rate is independent of the biomass concentration. As both communities reached a pseudo steady state, differences in biomass concentration are therefore related to compound concentrations and cultivation conditions. Here, it was found that changing the pH significantly affects the product ratios, biomass yield and catabolic rates.

### Weak Acid Uncoupling Causes Decreased Biomass Yield on Ethanol

In absence of product inhibition, enrichments in SBRs select for maximal growth rates and full substrate consumption. In this study, stably performing microbial communities were enriched in SBR systems but substrate limitation was never encountered. Therefore, it is likely that the functional differences observed between the two enrichments were related to the inhibitory effects of the fermentation products. Two main mechanisms of toxicity of the elongated products can be distinguished (Candry et al., 2018, 2020; Roghair et al., 2018). First of all, protonated short- and medium chain carboxylic acids induce ATP consumption for the extrusion of protons and dissociated acids (Herrero, 1983). This is referred to as weak acid uncoupling. The effect of weak acid uncoupling is equal for butyric and caproic acid due to their very similar dissociation constants (pK_*a*_). Secondly, medium chain carboxylic acids can dissolve in the cell membrane both in their protonated and unprotonated forms and interfere with membrane integrity and fluidity (Jarboe et al., 2013). This increases uncoupling effects by increasing membrane permeability and inherently reducing cellular efficiency (Desbois and Smith, 2010).

Decreasing the culture pH increases the relative amounts of protonated acids and thereby amplifies weak acid uncoupling. At pH 5.5, a significantly lower biomass yield was obtained than at pH 7.0 (Table 1). The biomass yield on ethanol at pH 7.0 of 0.11 ± 0.02 Cmol_*x*_ mol^–1^ was comparable to earlier reported biomass yields at neutral pH of 0.11 Cmol_*x*_ mol^–1^ (Candry et al., 2018) and 0.07–0.13 Cmol_*x*_ mol^–1^ (Kenealy and Waselefsky, 1985). The yield at pH 5.5 (0.05 ± 0.01 Cmol_*x*_ mol^–1^) was closer to the yield of 0.06 Cmol_*x*_ mol^–1^ reported by Thauer et al. (1968) in batch experiments with uncontrolled pH. The decrease in biomass yield is therefore likely to be related to toxicity through weak acid uncoupling.

### Caproic Acid Toxicity Steers the Balance Between Substrate Consumption and Product Inhibition

If weak acid uncoupling was the only mechanism of toxicity at play, both butyrate and caproate would cause a similar additional burden to the cells. The shift from caproate to butyrate production at pH 5.5 was therefore either arbitrary, or related to another mechanism of MCCA toxicity as the elongation of butyrate to caproate retains the same amount of acid equivalents. We hypothesize that the change in product spectrum is a protection mechanism against additional energy losses due to caproate dissolution in the cell membrane. In *Saccharomyces cerevisiae*, carboxylic acid toxicity was found to increase with increasing carbon chain length when comparing caproic, caprylic and decanoic acid (Liu et al., 2013). Likewise, decanoic acid becomes completely inhibitory at half the inhibitory concentration of caproic and caprylic acid in *Escherichia coli* (Royce et al., 2013). Furthermore, the inhibitory concentration of caproate is much lower than the inhibitory concentration of butyrate at neutral pH (Candry et al., 2018). This confirms that caproate, even in its dissociated form, has a stronger inhibitory effect than butyrate, likely related to the carbon chain length. Experimental validation is required to further elucidate the effect of the different mechanisms of toxicity of caproate on the product distribution in chain elongating communities.

By exchanging 50% of the culture broth with fresh medium at the beginning of an operational cycle, product and biomass concentrations are halved. The inhibitory effects of chain elongation products are therefore reduced. Throughout the cycle the product and biomass concentrations increased, which resulted in decreasing biomass-specific catabolic rates. This was reflected in largely stable hydrogen production rates during each operational cycle, despite strongly varying biomass concentrations (Figure 3). Various studies have shown that growth- and production rates are linearly affected by caproic acid toxicity, whereas butyric acid has a toxicity limit (Royce et al., 2013; Candry et al., 2018; Roghair et al., 2018). Therefore, the decreasing biomass-specific catabolic rate likely depended largely on the caproic acid concentration. Stable hydrogen production rates and incomplete substrate conversion at both pH confirmed that there is a delicate balance between substrate consumption and product inhibition. The apparent steady states were therefore governed by both the imposed retention time and the actual substrate and product concentrations. High concentrations of MCCAs impeded full substrate consumption and additional biomass growth. This underlines the relevance of bioreactor systems with integrated product removal for reaching complete substrate conversion and high MCCA production rates (Kucek et al., 2016).

### Higher q_*EtOH*_ at pH 5.5 Shows That Catabolic Overcapacity Is a Strategy for Dealing With Product Toxicity

As both enriched cultures performed stably, the stoichiometric and kinetic parameters can be compared. The more stringent environment at pH 5.5 led to a decrease in biomass yield and a shift in product spectrum. Surprisingly, the biomass-specific ethanol consumption rate was twice as high. This implies that (1) a higher catabolic capacity is needed at pH 5.5 and that (2) this available capacity could not be invested in increasing the growth rate at pH 7.0. Possibly, a higher catabolic rate could not effectuate a higher growth rate at pH 7.0 because the culture was already growing at μ^*max*^. However, the average growth rate in our systems was 0.058 h^–1^, which is significantly lower than μ^*max*^-values reported in literature (0.1–0.24 h^–1^) for *C. kluyveri* but comparable to growth rates (0.05–0.09 h^–1^) of communites enriched from anaerobic digester sludge in batch bottles (Weimer and Stevenson, 2012; Candry et al., 2018, 2020). Alternatively, faster growth might have been inhibited by relatively large amounts of fermentation products, leading to a trade-off between additional energy generation and maintaining endurable product concentrations. Considering the higher extent of weak acid uncoupling at mildly acidic pH, the higher catabolic flux might have been exploited for balancing increased non-growth-related ATP demands. A similar effect was seen when propionic acid was added to a culture of *Leptospirillum ferrooxidans* (Kleerebezem and Van Loosdrecht, 2008). A flexible q_*s*_^*max*^ might therefore be employed as a microbial strategy for dealing with product inhibition.

### The Advantages of SBRs as a Tool for Microbial Community Characterization

Both SBR and chemostat systems have been used as a tool for studying mixed culture physiology and for creating a selective pressure toward certain characteristics (Temudo et al., 2007, 2009; Stouten et al., 2019; Rombouts et al., 2020). In the absence of storage polymers, SBR cultivation leads to a selective pressure on growth rate, whereas chemostat cultivation selects for substrate affinity. Even though both cultivation strategies provide insightful data, this work once again shows that SBRs prove to be more suitable for kinetic parameter identification of microbial communities (Strous et al., 1998). Chemostat studies overlook the information that dynamic cultures give. Furthermore, when operating an SBR the effects and the order of effects of a perturbation become clear almost instantaneously. In future work, this allows the identification of the response of an established culture on changing process conditions, which is valuable for the generation of models upon which process design strategies can be based. Finally, every cycle in a functionally and taxonomically stable SBR is a biological replicate, improving the statistical significance of a stable culture. SBR cultivation can therefore be used to shed light on various aspects of chain elongating communities. For instance, the interactions between chain elongating organisms and methanogens have been studied to a limited extent and the effect of methanogen presence on chain elongator performance has not been quantified (Candry and Ganigué, 2021). SBR cultivation would allow for identifying the effect of the presence of methanogens in the microbial community on chain elongating activity. Furthermore, growth-rate based selective pressure could lead to alternative strategies to prevent competition for substrate while simultaneously allowing high-rate MCCA production. Alternatively, integrated SBR systems could be used for the optimization of process intensification strategies, such as in-line product extraction (Agler et al., 2012; Gildemyn et al., 2017) and simultaneous syngas fermentation and chain elongation (Diender et al., 2019).

In conclusion, SBR studies prove to be valuable for both fundamental characterization of microbial communities and for studying process optimization strategies. Beside substrate uptake kinetics, SBRs can be used for the fundamental understanding and mathematical description of the effect of product toxicity and changing ethanol-to-acetate ratios on chain elongating organisms in dynamic environments. Omics techniques in combination with metabolic modeling could be used to identify labor division in microbial communities fed with a complex substrate and gain a deeper understanding of the effect of environmental conditions on the metabolic flux though microbial communities (Scarborough et al., 2018). Lastly, integrated reactor setups can be exploited for testing the industrial potential of different cultivation strategies.

## Data Availability Statement

The datasets presented in this study can be found in online repositories. The names of the repository/repositories and accession number(s) can be found in the article/Supplementary Material.

## Author Contributions

MTA performed the experiments. MTA and GRS analyzed the data. All authors wrote the manuscript.

## Conflict of Interest

The authors declare that the research was conducted in the absence of any commercial or financial relationships that could be construed as a potential conflict of interest. The Guest Associate Editor RG, declared a past collaboration with one of the authors RK.
