# Supplementary material for: Product Inhibition and pH Affect Stoichiometry and Kinetics of Chain Elongating Microbial Communities in Sequencing Batch Bioreactors
Source: Front Bioeng Biotechnol. 2021 Jun 21;9:693030. doi: 10.3389/fbioe.2021.693030 (PMC8256265; doi:10.3389/fbioe.2021.693030)
Supplement: Supplementary file 1 [file Data_Sheet_1.docx]

Supplementary Material

Supplementary Table 1. Average steady state substrate and product concentrations at the start and end of the cycle and final biomass concentrations and hydrogen production. End of cycle concentrations were measured in the fermentation broth and cycle start concentrations were calculated taking into account the exchange ratio of 0.5. The measurements from samples in the operational cycles between 60 and 100 were taken to calculate the average start and end concentrations, as the systems performed stably during this period of time. Carbon- and electron balances were calculated assuming a normalized stoichiometry of C_1.0_H_1.8_O_0.5_N_0.2_ for biomass.

|  | pH 7.0  *start* | pH 7.0  *end* | pH 5.5  *start* | pH 5.5  *end* |
| --- | --- | --- | --- | --- |
| **Concentration (mmol L^-1^)** |  |  |  |  |
| *Ethanol* | 172 | 93±11 | 201 | 150±9 |
| *Acetate* | 24 | 6±3 | 25 | 7±3 |
| *Butyrate* | 12 | 25±5 | 24 | 48±6 |
| *Caproate* | 25 | 49±2 | 7 | 14±2 |
| *Biomass* |  | 18.0±2.4 |  | 5.2±0.4 |
| *Hydrogen produced (mmol)* |  | 25.5 |  | 14.2±1 |
| **Balances** |  |  |  |  |
| *Carbon* |  | 106% |  | 104% |
| *Electrons* |  | 102% |  | 100% |

# Biological respiration rate determination

The biological respiration rates were reconstructed by combining Particle Filter simulations with a physicochemical model (Stouten et al., 2021). Each randomized particle represents a possible state of the system (i.e.: respiration rates, where a system modeled by 100.000 particles represents 100.000 unique bioreactor simulations). During each predication step the physicochemical model calculates for each particle how it would influence the whole system (e.g.: the gas concentrations in the off-gas). When measurements are available (i.e.: intermittent off-gas measurements), the particles that statistically overlap with the measurements are preserved, and the discarded particles are replaced by copies of the preserved particles.

As the bioreactors are operated under dynamic conditions (sequencing batch), it is expected that the respiration rates of the biomass change throughout a cycle. Therefore, during each prediction step each particle can change its respiration rates. This change in respiration is a normally distributed around 0, with a specific variance. This variance is an input to the model. If strong changes in biological respiration rates are expected the variance needs to accommodate for this, alternatively if the variance is chosen too large then a significant fraction of the particles will reach unlikely predictions in between the measurements.

The covariance matrix ensures that the obtained predictions are in a biologically relevant range and are therefore dependent on the system that is being studied. Here, fermentative organisms that do not fix nitrogen were studied, but nitrogen is present in the in-gas. Therefore, the variance in the biological nitrogen respiration rate equals zero. Methanogenic organisms were present in such low abundance during stable bioreactor operation that the methane respiration rate, and the change therein, was at least an order of magnitude smaller than the hydrogen respiration rate. At pH 5.5 a significantly lower biomass concentration was observed, lowering the total biological capacity in the system. The process covariance was limited accordingly.

The process covariance matrix (mol^2^ s^-2^), determining how fast the biological respiration rates can change, was defined as follows:

$$\begin{aligned} U_{k, pH 7.0}\mathcal{=N}\left( \left( \begin{aligned} \begin{matrix} 0 \\ 0 \\ 0 \end{matrix} \\ 0 \end{aligned} \right),diag\left( \begin{aligned} \begin{matrix} 1 \\ 1 \\ 1 \end{matrix} \\ 1 \end{aligned} \right)\cdot\left( \begin{aligned} \begin{matrix} 0 \\ 2\cdot{10}^{-9} \\ 2\cdot{10}^{-9} \end{matrix} \\ 2\cdot{10}^{-10} \end{aligned} \right) \right) \end{aligned}$$

$$U_{k, pH 5.5}\mathcal{=N}\left( \left( \begin{aligned} \begin{matrix} 0 \\ 0 \\ 0 \end{matrix} \\ 0 \end{aligned} \right),diag\left( \begin{aligned} \begin{matrix} 1 \\ 1 \\ 1 \end{matrix} \\ 1 \end{aligned} \right)\cdot\left( \begin{aligned} \begin{matrix} 0 \\ 1\cdot{10}^{-9} \\ 5\cdot{10}^{-10} \end{matrix} \\ 1\cdot{10}^{-10} \end{aligned} \right) \right)$$

With U_k_ representing the covariance matrix for nitrogen, hydrogen, carbon dioxide, and methane uptake rates.

As the internal state estimation of the biological system is based on measurement data, the uncertainty in these measurements has to be taken into account in order to determine the accurate range of the estimation of the internal state at each timepoint. The uncertainty of the measurement depends on the accuracy of the mass spectrometer, which depends on the concentrations and is different for the different gaseous species that are measured. This noise covariance for the off-gas (N_2_, H_2_, CO_2_, CH_4_) measurements (expressed as ppm^2^) is expressed in the covariance matrix of the measurements:

$$R_{k}\mathcal{\sim N}\left( \left( \begin{aligned} \begin{matrix} 0 \\ 0 \\ 0 \end{matrix} \\ 0 \end{aligned} \right),diag\left( \begin{aligned} \begin{matrix} 1 \\ 1 \\ 1 \end{matrix} \\ 1 \end{aligned} \right)\cdot\left( \begin{aligned} \begin{matrix} 30000 \\ 2500 \\ 2500 \end{matrix} \\ 250 \end{aligned} \right) \right)$$

With R_k_ representing the covariance of head space gas mole fraction (N_2_(g), H_2_(g), CO_2_(g), CH_4_(g).

**Supplementary Figure 1.** Measured (red dots) and modeled (grey crosses) off-gas fraction of hydrogen and resulting hydrogen uptake rate (blue dots) in mol s^-1^ of cycle 90-96 of the enrichment at pH 7.0. A negative uptake rate represents a positive production rate.
